# The Ethics of Gene Therapy for Sickle Cell Disease

**DOI:** 10.7759/cureus.81037

**Published:** 2025-03-23

**Authors:** Amie Patel, Alice Kuo

**Affiliations:** 1 Division of Medicine-Pediatrics, David Geffen School of Medicine, University of California Los Angeles, Los Angeles, USA

**Keywords:** ethic consideration, gene therapy, informed consent, neurocognitive impairment, sickle cell disease: scd

## Abstract

Gene therapy for sickle cell disease (SCD) can drastically transform an individual's life and decrease the disease burden, but there are several ethical dilemmas associated with it. This editorial examines the various challenges, including the quality of informed consent, patient selection criteria, and equitable access. While gene therapy offers significant promise, addressing the ethical concerns surrounding it is crucial to ensure responsible implementation and equitable care for individuals with SCD and their communities.

## Editorial

My sister was born with sickle cell beta thalassemia zero, a condition that brought unimaginable pain into her life. Once she started high school, we witnessed her enduring the agony of vaso-occlusive pain crisis regularly. Each one of these crises was a harsh reminder of her ongoing suffering. With all this pain that she had to suffer, my parents took to any glimmer of hope to find a way to make the suffering go away. At 17, my sister underwent an unrelated donor bone marrow transplant in the hopes of finding a cure. That hope and optimism that she would be “cured” outweighed any fear of the hypothetical risk that loomed ahead. Once my sister underwent the transplant, she switched one chronic disease state for another as she battled unrelenting graft versus host disease. Despite her courageous fight, she passed away two years after she received the transplant.

In the late 1990s/early 2000s, bone marrow transplantation was hailed as a beacon of possibility and represented great promise for finding an ultimate cure for sickle cell disease (SCD). However, as more and more individuals underwent bone marrow transplantation for treatment and more complications occurred, increased hesitation regarding recommending transplants started to emerge. This uncertainty around bone marrow transplant is reflected in the 2014 guidelines issued by the National Heart, Lung, and Blood Institute (NHLBI), acknowledging that additional research is required to determine the optimal transplant regimen [1].

This overly optimistic view on allogenic bone marrow transplants raises questions regarding the excitement surrounding the two gene therapies for SCD (Lyfgenia and Casgevy) that were FDA-approved on December 8, 2023. Both of these gene therapies show significant promise with this life-transformative treatment that profoundly reduces or eliminates the risks for vaso-occlusive pain crises. Additionally, both studies have shown laboratory markers consistent with less hemolysis after gene therapy. However, early trials have already demonstrated some possible toxicities, including individuals developing acute myeloid leukemia and myelodysplastic syndrome with alpha deletions, which must be considered when evaluating the overall benefit-risk profile of these therapies [2-3].

The excitement and promise about what these treatments can offer should be balanced with a sense of realism about all the unknowns. For one, we do not fully know if this is truly a cure as we only have about six years’ worth of data and do not know if individuals will continue to have the same beneficial effects in the long term. Additionally, there are more questions and a lack of consensus when it comes to gene therapy. Who is the ideal candidate for gene therapy and who should be excluded? Who is responsible for following these patients for the FDA-mandated 15 years of long-term follow-up? Is it the general hematologist, the transplanter, or their primary care provider? Who is monitoring off-target effects and for how long? What is considered a success? How should we treat individuals who continue to have SCD complications, such as ongoing hemolysis or pain? Does a degree of hemolysis persist even after gene therapy that can still lead to organ dysfunction? Who will have access to this therapy? These are just a few of the many questions that need to be addressed.

Although these therapies are now FDA-approved, there is no standard informed consent process. Hence, it is difficult to determine if patients are fully informed about the ongoing uncertainties. Many individuals with SCD have executive functioning and working memory deficits that are underdiagnosed [4]. It is vital to evaluate the cognitive capacity of each individual before a valid informed consent process, but this practice is not standard in this patient population [5]. Additionally, with all the unknowns in terms of long-term side effects, there is an ethical question as to whether pediatric patients should be included in gene therapy procedures and if there should be a standard assent process for minors. On the other hand, not offering early gene therapy to prevent further organ damage from severe SCD could be withholding potentially life-altering treatment.

Additionally, SCD affects about 100,000 people in the United States with more than 90% being African American or Black [6]. This population has historically suffered from significant health disparities and reduced equity/access to healthcare resources. Is it an ethical proposition to allocate healthcare funds to try to increase access to very costly gene therapy while most SCD patients have limited access to basic preventive care from a trained sickle cell specialist to help modify their disease? We need to understand what the patients truly desire as we approach them with this transformative therapy. In the 1990s, the National Association of the Deaf called the FDA’s decision to approve cochlear implants in children “unsound scientifically, procedurally, and ethically” as they viewed the surgery as “invasive for defenseless children" [7]. Since 2014, they have adjusted the opinion; however, it emphasized the imperative of having the communities speak in terms of the ethics regarding this transformative therapy.

Gene therapy offers a lot of promise. As a physician and a sister of someone with SCD, I am excited about the possibility that gene therapy can change lives dramatically for individuals suffering from SCD and their families. The hope for a transformative treatment brings the optimism necessary to keep fighting and looking for better ways to treat SCD. Was my sister’s bone marrow transplant worth it? I do not know. However, I do have the utmost respect for all of my sister’s transplanters as I can imagine the life of suffering she would have endured without it. As we register substantial advancements in our therapies, from an ethical standpoint, a comprehensive framework should be established that includes standardized informed consent processes, cognitive assessments tailored to the SCD population, and community engagement to ensure patient-centered care. This framework must also address equitable access and long-term monitoring to maintain ethical integrity in the evolving landscape of gene therapy. We owe it to every patient and family to ensure that our pursuit of progress is grounded in ethical integrity.
